# Internet-based psychotherapy in children with obsessive-compulsive disorder (OCD): protocol of a randomized controlled trial

**DOI:** 10.1186/s13063-022-06062-w

**Published:** 2022-02-21

**Authors:** Annette Conzelmann, Karsten Hollmann, Anna Haigis, Heinrich Lautenbacher, Verena Bizu, Rehan App, Matthias Nickola, Gunilla Wewetzer, Christoph Wewetzer, Tord Ivarsson, Norbert Skokauskas, Lidewij H. Wolters, Gudmundur Skarphedinsson, Bernhard Weidle, Else de Haan, Nor Christian Torp, Scott N. Compton, Rosa Calvo, Sara Lera-Miguel, Annika Alt, Carolin Sarah Hohnecker, Katharina Allgaier, Tobias J. Renner

**Affiliations:** 1 Department of Child and Adolescent Psychiatry, Psychosomatics and Psychotherapy, University Hospital of Psychiatry and Psychotherapy, Osianderstr. 14-16, 72076 Tübingen, Germany; 2 PFH – Private University of Applied Sciences, Department of Psychology (Clinical Psychology II), Göttingen, Germany; 3 Section for Information Technology, University Hospital Tübingen, Tübingen, Germany; 4 Freelance Software Developer, Reutlingen, Germany; 5 Child and Adolescent Psychiatry and Psychotherapy, Clinics of the City of Cologne, Cologne, Germany; 6 Institute of Neuroscience and Physiology, University of Gothenburg, Gothenburg, Sweden; 7 Regional Centre for Child and Youth Mental Health and Child Welfare Faculty of Medicine and Health Sciences, Trondheim, Norway; 8 Department of Child and Adolescent Psychiatry, Academisch Medisch Centrum Universiteit van Amsterdam, Amsterdam, The Netherlands; 9 Faculty of Psychology, University of Iceland, Reykjavik, Iceland; 10 Academic Center for Child and Adolescent Psychiatry, Amsterdam, The Netherlands; 11 Centre for Child and Adolescent Mental Health, Eastern and Southern, Division of Mental Health and Addiction, Oslo, Norway; 12 Division of Mental Health Services, Akershus University Hospital, Lørenskog, Norway; 13 Duke Child and Family Study Center, Durham, USA; 14 Department of Child and Adolescent Psychiatry and Psychology, Hospital Clínic de Barcelona, Barcelona University, CIBERSAM, Barcelona, Spain

**Keywords:** Internet psychotherapy, App, Obsessive-compulsive disorder, Randomized controlled TRIAL, Children

## Abstract

**Background:**

Obsessive-compulsive disorder (OCD) in children can lead to a huge burden on the concerned patients and their family members. While successful state-of-the art cognitive behavioral interventions exist, there is still a lack of available experts for treatment at home, where most symptoms manifest. Internet-based cognitive behavioral therapy (iCBT) could overcome these restrictions; however, studies about iCBT in children with OCD are rare and mostly target computerized self-help resources and only email contact with the therapist. Therefore, we intended to build up and to evaluate an iCBT approach for children with OCD, replacing successful elements of traditional in-office face-to-face CBT, with face-to-face teleconferences, online materials, and apps.

**Methods:**

With the help of a pilot feasibility study, we developed the iCBT consisting of 14 teleconference sessions with the child and parents. The sessions are supported by an app assessing daily and weekly symptoms and treatment course completed by children and parents. Additionally, we obtain heart rate and activity scores from the child via wristbands during several days and exposure sessions. Using a waiting list randomized control trial design, we aim to treat and analyze 20 children with OCD immediately after a diagnostic session whereas the control group of another set of 20 OCD patients will be treated after waiting period of 16 weeks. We will recruit 30 patients in each group to take account for potential dropouts. Outcomes for the treatment group are evaluated before randomization (baseline, t0), 16 weeks (end of treatment, t1), 32 weeks (follow-up 1, t2), and 48 weeks after randomization (follow-up 2, t3). For the waiting list group, outcomes are measured before the first randomization (baseline), at 16 weeks (waiting list period), 32 weeks (end of treatment), 48 weeks after the first randomization (follow-up I), and 64 weeks after the first randomization (follow-up II).

**Discussion:**

Based on our experience of feasibility during the pilot study, we were able to develop the iCBT approach and the current study will investigate treatment effectiveness. Building up an iCBT approach, resembling traditional in-office face-to-face therapy, may ensure the achievement of well-known therapy effect factors, the acceptance in both patients and clinicians, and the wide distribution within the health system.

**Trial registration:**

ClinicalTrials.govNCT05037344. Registered May 2019, last release August 13th, 2021.

## Background

The mental health of children and adolescents is a high-priority area and there is a need for effective and easily accessible treatments. Obsessive-compulsive disorder (OCD) is characterized by fear, worry-provoking intrusive thoughts, and repetitive behaviors that reduce the associated distress. Without treatment, OCD is a chronic and highly impairing disorder, with a population prevalence rate between 1 and 3% [1].

Cognitive behavioral therapy (CBT) is a highly effective treatment for OCD in children and adults [2, 3]. During exposures with response prevention (ERP), patients are confronted with their intrusive thoughts and preceding situations; patients learn to stop their repetitive behaviors followed by a natural decline in distress and learning experience that intrusive thoughts are harmless. Unfortunately, there is limited availability of therapists adequately trained in this specific CBT procedure for OCD. In addition, ERP is hardly used during CBT because of practicability in everyday therapeutic life, or negative assumptions of the therapists, just to mention a few [4]. Apart from this, one major obstacle is also the fact that access to appropriate treatment is particularly difficult for children and adolescents in rural areas due to fewer psychotherapists being locally available [5. Accordingly, too few children with OCD get appropriate help [6]. Medication is much more accessible than CBT [7]. Another problem within face-to-face settings is that the psychotherapist often acts as security signal preventing a strong realistic symptom provocation. In addition, symptoms are especially present at home and, therefore, therapy should be more efficient, when these symptoms are treated in the corresponding triggering environment. OCD patients themselves also sometimes refuse psychotherapy due to avoiding behavior, fear of stigmatization, geographical distance to experts and time restrictions, especially in rural areas [8]. Patients, therefore, often suffer from a long delay until getting effective treatment. Late treatment onset is critical as the longer a patient is without effective treatment, the more likely their symptoms are to become chronic [9]. Accordingly, internet-based treatments with CBT experts could address an important gap in availability of treatments [10]. Moreover, due to the Covid-19 pandemic, psychotherapy was only possible via teleconferences which pushed acceptance of psychotherapists and patients forward for this new method more than ever before. iCBT could be especially attractive for pediatric patients as 90–96% of young people aged 12–24 have good internet accessibility and their ability to use new media platforms should be high [11]. It can be also cost-effective [12]. Furthermore, it may even be possible to reach and treat more patients across the country. The inclusion of parents in the therapeutic process is also more practical and easier via internet, as compared to traditional face-to-face approaches, as they can be part of the process no matter their location, as long as they have an internet connection.

Studies on iCBT approaches are encouraging, especially regarding anxiety disorders or depression. The findings of studies, reviews, and meta-analyses in patients (mostly adults, but also in children) suggest that CBT programs with computerized elements (e.g., online material, email contacts) are just as effective as CBT from traditional face-to-face therapies [13–20]. There are also a few studies reporting promising results on patients with OCD [21]. Studies using self-help iCBT in adults or children with OCD revealed therapy success [22–25]. Other studies in adults and children with OCD used therapist-assisted text-message-based iCBT approaches which were found to be very effective, although therapists in some of these studies were not clinical experts [11, 26–30]. There are also results revealing long-term efficiency and cost-effectiveness [16, 31]. Concurrently, one study compared therapist-guided iCBT, unguided iCBT, and traditional face-to-face therapy in adults with OCD to get more information about the requirement of therapist involvement, which may be an important factor for a successful therapeutic intervention [32]. Reviews dealing with self-help interventions in OCD suggest that the more a therapist is involved in interventions, the more likely it is that there are successful treatment outcomes [33, 34]. There have only been a few iCBT approaches using telephone conferences between patients and therapists for OCD, and the results are promising. However, due to small samples sizes, treatment by less trained therapists, or often a lack of control groups in these studies [5, 35–37], further research is needed; hence, the present study aims to address some of these caveats.

Although iCBT approaches exist, it is evident that more studies on specific disorders and more studies in children and their families are needed. Therapist contact seems to be an important moderator and may be especially important for long-term benefits and patient adherence [15]. Nevertheless, most existing iCBT programs either use self-help elements, lack a control condition, lack visual contact with the therapist, or provide therapy only by trained practitioners rather than psychotherapy experts. An approach replacing traditional face-to-face CBT with online elements, such as teleconferences, whilst following similar protocols to traditional settings, would increase acceptability and the distribution of these tools in the health system. Finally, and most importantly, such an approach would contribute to secure and improve the health of the patients. Enriching such an approach with further benefits of new media, such as an app-based assessment of daily symptoms, avoidance behavior, daily hassles, and functioning or therapeutic homework by apps may additionally increase the health benefit over traditional face-to-face psychotherapies as well as the self-efficacy of the patients.

Therefore, we continuously met with international experts on OCD treatment to develop evidence-based ideas for an internet-teleconference-based psychotherapy manual and a supporting app. Together, with the IT department of the university hospital and an external programmer, we developed a data security concept and IT software to use these technologies and to transfer the data safely into internal databases. We already treated OCD patients with this approach in a pilot study and could show that patients appreciated our intervention. Therefore, we now want to go beyond feasibility and test the effectiveness of our treatment in a waiting list design. We hypothesize that symptoms decline over treatment in comparison to the waiting group that later will also benefit from the treatment. The long-term goal is to develop an effective and acceptable internet-delivered pediatric OCD treatment intervention which can also be generalized for the treatment of other disorders.

## Methods/design

### Subjects

The study has a parallel group design with an experimental group receiving treatment after baseline assessment and a waiting group receiving treatment after a waiting period of 16 weeks, comparably to the length of treatment. The planned allocation ratio is one expecting superiority in symptom reduction in the experimental group. Patients are recruited by licensed child and adolescent psychotherapists who work at the university hospital of Tübingen. A Google AdWords advertising and recruitment campaign is carried out in cooperation with the Department of Communication at the University Hospital Tübingen.

To deal with probable dropouts, we planned to enroll 30 children with OCD in the experimental group and 30 children with OCD in the waiting group with the aim to finally analyze 20 patients in each group. Including clinical considerations, this was a realistic sample size, we were able to achieve in a 3-year period. Sample size was planned according to the following considerations. As primary outcome, we considered the CY-Bocs score. Our primary assessment endpoint was the comparison at t1 post-treatment between the treatment and the waiting group. In the pilot study of our treatment protocol using a pre/post-assessment with *N* = 9 children with OCD [38], effect size was *d* = 2.02. Therefore, we knew, that our treatment protocol most likely will lead to symptom reduction. Effect size of another study comparing webcam-delivered CBT in 16 children with OCD with a waiting group of 15 children with an ANCOVA on post-treatment CY-Bocs scores, covarying baseline CY-BOCS scores, showed a significant effect of treatment with *η*^2^ = 0.36 [37]. A further study in adults with OCD [39], comparing face-to face exposure treatment (*n* = 20) with a waiting group (*n* = 16), revealed an effect size of *d* = 1.001 for the CY-Bocs comparison at post-treatment. Therefore, we estimated that the effect in our study as a comparison of CY-Bocs scores of an effective treatment in comparison to the waiting group at t1 will be high. We expected that there will be a significant difference comparing 20 patients with treatment with 20 patients in the waiting group at t1.

All participants and their parents provide informed consent. On the consent form, participants are asked to allow the use of their data also for future projects of the department and the study team. The consent forms are available from the corresponding author on request. The trial does not involve collecting biological specimens for storage. Patients will be recruited through established OCD outpatient units in Tübingen and Cologne (Germany), OCD Society, the Clinic’s homepage, schools, Google AdWords, an interview with an influencer suffering from OCD, and local psychiatrists and psychologists in Tübingen.

After a telephone interview and a first visit at our research department in Tübingen to verify the suitability for taking part in the study, and the face-to-face diagnostic session, participants will be randomized to the experimental or waiting list groups. Inclusion criteria will be children and adolescents (aged between 6 and 18) with a primary diagnosis obsessive-compulsive disorder according to DSM-5, a CY-BOCS score higher than 16 and at least one primary caretaker, German-speaking (child & caretakers), and a family home equipped with a broadband internet connection. Psychiatric comorbidities are allowed as long as the comorbid disorder does not have a higher treatment priority than OCD. Participants are excluded if they have an IQ below 70, do not speak or understand German, have a psychiatric comorbidity or suicidality that makes participation clinically inappropriate, or if they should be treated in the hospital. Medication is allowed if treatment was stable for 6 weeks before inclusion into the study and will then be taken during the trial. Furthermore, patients will be excluded in case of drug addiction or if the family seems to be severely psychologically burdened such that participation in the sessions and support of the children during the trial will not be possible. This will also be discussed in the steering board of our study team. While participating in the study, no other psychological treatment is allowed. If reporting side effects, or if wished by the patients, the patients will be excluded from the study and transferred to another more appropriate therapy option.

### Study procedure and design

The study and treatment will be conducted by the Department of Child and Adolescent Psychiatry, Psychosomatics and Psychotherapy Tübingen, Germany, a university hospital. The design of the study is open label for the therapists who will treat the patients and blinded for the diagnostician. Emergency unblinding will only happen for dropouts or severe cases. All procedures and the data security concept were evaluated by the Ethical Committee of the Medical Faculty of the University of Tübingen with the vote 639/2018BO1 dated 09/18/2018 (participant information, consent form, and data security concept are available from the authors upon request). All assessors who are involved in the study are trained psychotherapists (including a 3-year training after university studies of psychology/pedagogics). They also have profound training in diagnostics and statistical analysis. To maintain the scientific qualification, courses in Good Clinical Practice are to be completed regularly for all evaluators and study members.

In a telephone screening for eligibility by our study team (all experienced psychotherapists), participants are informed about the procedures. The enquiries received by the study team by phone or email will then processed by the treating therapists and contact will be made with the interested parties. If the inclusion criteria are met, an initial interview and a diagnostic appointment including introduction into the technical procedures will be arranged. Participants will get clinical questionnaires to fill out at home. Parental and children consent for study participation will be obtained in written form during the diagnostic appointments, which are conducted by a psychologist at the department face-to-face.

The participants will be assigned to the treatment or waiting group by block randomization according to the order of incoming calls. The randomization list is computer-generated and randomized to assign subjects to both groups in equal proportions. The randomization list was developed by our Institute for Clinical Epidemiology and Applied Biometry (IKEaB) with 8 blocks of 6 participants, consisting of *n* = 3 participants as waiting group and *n* = 3 participants starting treatment directly. The diagnostic expert will be blinded to the group condition. The assignment of the subjects will be pseudonymized by the randomization list, so that the diagnostician does not know which child is assigned to which group. The randomization list will be stored in a sealed envelope in the cabinet. After the patients are assigned to either the waiting or treatment group by block randomization according to the sequence of calls and inclusion in the study, they receive feedback on the allocation from a therapist from our study team. For this purpose, the therapist looks into the sealed envelope containing the order of the randomization list. This feedback is given either by telephone call or in person during the meeting on site at the clinic.

In the treatment condition, there are 14 weekly teleconference psychotherapy sessions lasting approximately 90 min each. Post-assessment will take place face-to-face. A total of 16 weeks after the treatment, there will be a first follow-up with clinical questionnaires that are sent to the families and a teleconference-based diagnostic session. A second follow-up will take place after another 16 weeks to further evaluate the effects and to enable comparing of the progress between treatment and waiting list group.

**Fig. 1:**
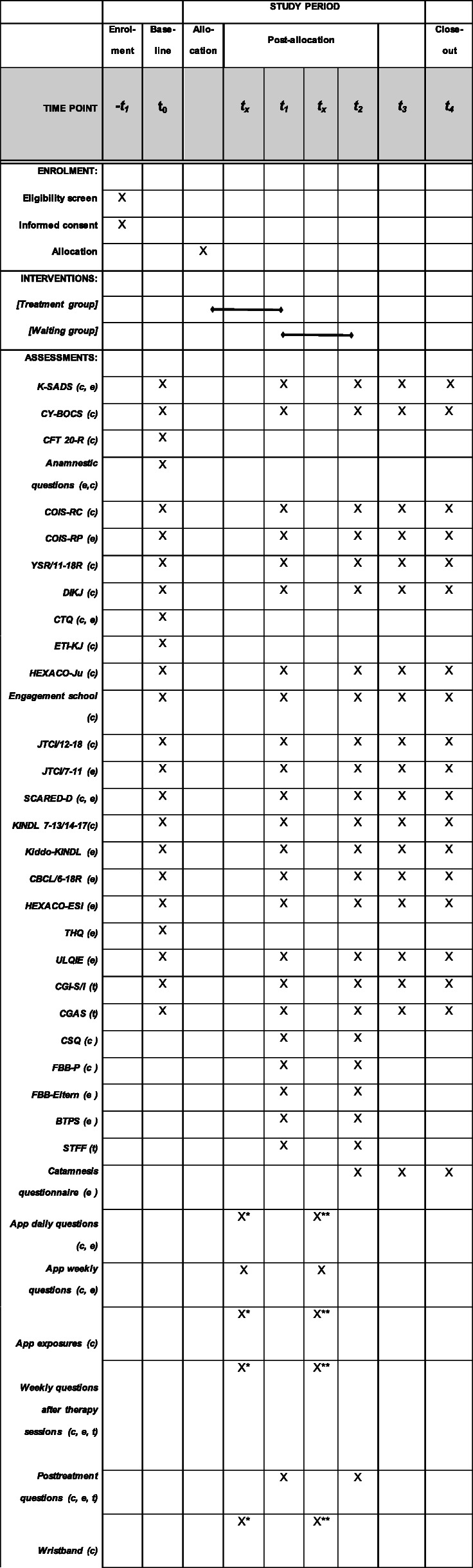
Schedule of enrolment, interventions, and assessments. Information about the diagnostic measures can be found in themanuscript text. Time points: -t1 = First contact and enrolment to the study; t0 = Baseline assessment; tx = Appassessments; t1 = After 16 weeks, Assessment at the end of intervention treatment group/begin treatment waiting group; t2= After 32 weeks, Follow-up I for treatment group/end of intervention waiting group; t3 = After 48 weeks, Follow-up IItreatment group/follow-up waiting group; t4 = After 48 weeks, Follow-up II waiting group. X means assessment in thetreatment and waiting group unless it is indicated that the assessment is only carried out in one specific group. TG=treatment group; WG = waiting group. p=assessed in parents, c = assessed in children, t =assessed in therapist. * only TG,** only WG

For the treatment group, outcomes will be evaluated before randomization (baseline, t0), after 16 weeks (end of treatment, t1), 32 weeks after randomization (follow-up I, t2), and 48 weeks after randomization (follow-up 2, t3). For the waiting list group, outcomes will also be measured before the first randomization (baseline, t0), at 16 weeks (waiting list period, t1), at 32 weeks (end of treatment, t2), at 48 weeks after the first randomization (follow-up I, t3) (compare Fig. 1), and at 64 weeks after the first randomization (follow-up II, t4).

Adherence will be supervised by questionnaires before and after each session, by discussions with the therapist and by continuous supervision of homework. Participants get written instructions for all technological procedures. Any missing data, homework, and other problems concerning the intervention are discussed within the study group and patients are then approached by strategies to increase compliance.

Participant retention is ensured by a good contact between all team members and the participants. If participants do not complete follow-up assessments, they are reminded by the study team or their therapist several times. Participants are also encouraged to follow the planned interventions; however, if other interventions might be more appropriate, they will be transferred to other treatment options. The patients agree to participate in the study and the associated follow-up interviews by means of the consent forms. Intrinsic motivation is triggered by the timely participation in the therapy by experts within the framework of the study and the reduction of the obsessive-compulsive symptoms through the treatment in the study, and extrinsic motivation is the encouragement by the diagnostician to carry out the follow-up interviews. There is no financial compensation for the patients.

The documentation of the follow-ups including questionnaires will be recorded manually in the respective patient folders and kept locked in the cabinets in the clinic. If participants do not at all adhere to the protocol, are not motivated, or want to quit the study, they will drop out of the study; however, this will be the last option and motivational work will be done in advance. All planned outcomes will, nevertheless, be assessed for dropouts. A digital list of dropouts is kept within the study team and we note the reason for their dropout. Information about non-compliance, problems, or harms are continuously documented during the trial after every treatment session.

Data collection is monitored by our study team who meets every week. If problems with data collection become obvious, the team decides together about required next steps to ensure data quality, e.g., telephone call with the participants to encourage data completion. There is no external supervision of the data. As we use a low-risk intervention, there will be no trial auditing. As mentioned, the project team meets every week to discuss trial conduct.

There are no planned interim analyses that might lead to trial finalization. Trial would be stopped only if the team observes severe clinical reasons to stop the trail. These are, however, not expected because of our preliminary study and the fact that treatment is based on state-of-the art therapy methods. Data are in part only evaluated when needed, for example to show first preliminary results in presentations on conventions. If there are any changes to the protocol, these are discussed within the study team and the sponsor and changes are documented. If the protocol must be changed, the trials registry ClinicalTrials.gov will be updated. Only the study team has access to the final dataset.

Results will be disseminated in scientific papers, presentations in conventions, or within the university hospital or public media. Additionally, the funder will be informed about the results.

### Questionnaires and interviews

Figure 1 depicts the time points of measurement and source of all dependent variables.

#### Primary outcome

The Children’s Yale-Brown Obsessive Compulsive Scale (CY-BOCS) [40] will be used for the assessment of OCD symptoms and treatment response. The CY-BOCS is a clinician-administered instrument that evaluates obsessions and compulsions. The primary assessment endpoint is the comparison between the treatment and waiting group at t1.

#### Secondary outcomes

The Child Obsessive-Compulsive Impact Scale (COIS-RC) is a 33-item self-report questionnaire to the impact of OCD symptoms on the psychosocial functioning of the children [41]. With a *sociodemographic questionnaire*, we will assess basic variables such as age, sex, course of the disorder, family situation, and school. The Schedule for Affective Disorders and Schizophrenia for School-Age Children Present and Lifetime Version (K-SADS-PL) [42] as a semi-structured clinical interview will be used for diagnostic assessments of a wide variety of psychiatric disorders. Clinical Global Impressions-Severity (CGI-S, SCI-I) is a clinical 7-point rating scale of symptom severity [43]. *The Youth Self Report (YSR/11-18R)* is a 112-item 3-point scale completed by the children themselves and addresses a variety of behavioral and emotional problems [44]*. Child Behavior Checklist* (CBCL/6-18R), the CBCL, is a 113-item 3-point questionnaire for parents that assesses a wide range of child behavioral and emotional problems [45]. Screen for Child Anxiety Related Emotional Disorders (SCARED): The SCARED assesses DSM-IV anxiety symptoms [46]. Questionnaire for Measuring Health-Related Quality of Life in Children and Adolescents (KINDL/Kiddo KINDL) is a 24-item 5-point scale self-report questionnaire for children and parents to quality of life [47]. With the *Lebensqualitäts-Inventar für Eltern chronisch kranker Kinder* (ULQIE), we will assess life quality of parents related to the impairment through the illness of the child [48]. Culture-free test (CFT-20-R) to assess cognitive functioning is used as a test for intelligence [49]. The *Depressionsinventar für Kinder und Jugendliche* (DIKJ) will measure depressive symptoms in the children. It has 26 items and a 4-point scale [50]. We assess emotional, cognitive, and behavioral e*ngagement at school* with 5 items each filled out by the children and adolescents [51].

#### Other outcomes

The Junior Temperament and Charakter Inventar (JTCI/12-18R/JTCI/7-11R) assesses temperament characteristics with 103/86 items and a 5-point scale [52]. To assess the core personality traits of children until 12 years, the parents will fill out the HEXACO- Elementary School Inventory (HEXACO-ESI). Adolescents (12 years and older) complete according items from a middle school inventory themselves (HEXACO-JU) based on the HEXACO model of personality factors [53]. For another study, we will also assess trauma questionnaires to answer trauma-related questions: The *Childhood Trauma Questionnaire* (CTQ) as a 5-point scale and 28 items measures trauma experience such as sexual abuse or emotional neglect [54]. Furthermore, we will assess the *Essener Trauma Inventar für Kinder und Jugendliche* (ETI-KJ) for posttraumatic symptoms [55] as well as the *Trauma History Questionnaire* (THQ) as a 24-item measure of traumatic events [56].

#### Session questionnaires

After each session, we will obtain information about the following: (a) impairment through specific OCD symptoms, (b) questions about satisfaction with the session and (c) the patient-therapist relationship, (d) questions about homework compliance and technical problems, (e) information about missed sessions, (f) deviations from the manual, and (g) session length. With a post-experimental questionnaire, we will ask for satisfaction about different elements of the therapeutic intervention, such as (a) manual, (b) technique, (c) patient-therapist relationship, (d) improvement, (e) problems, (f) preference over traditional therapy approaches, and (g) decisions about useful therapy elements. These questions were also partially derived from questionnaires about treatment fidelity, acceptability, integrity, and engagement such as Session Integrity Checklist (SIC) used by Kazdin and colleagues [57], the Manual Rating Form (MRF developed by Kendall and colleagues [58], a Therapist Feedback Form (STFF), barriers to treatment scale (BTPS [59]), a consumer satisfaction questionnaire (TAI, [60]), and the Client Satisfaction Questionnaire-8 (CSQ-8 [61]). We will also collect information to premature dropouts and ask participants whether they agree to fill out our post-experimental questionnaires. The therapist will also provide data in post-experimental questionnaires.

### Apps

We will use two apps for the project. The first app is an app with specific questions about the severity of children’s OCD and evaluations of the day or week. We will assess children and their parents with questions to the children’s OCD symptoms and impairment, avoiding behavior, mood, quality of the day, and daily hassles every day (in the evening). Furthermore, once a week, we will obtain in children’s and parents’ information about symptom development. Finally, the app prepares and evaluates exposures as well as levels of distress before, during, and after the exposures as well as avoiding behavior. Data of this app will be used for the therapy sessions and is discussed with the families. The second app will be a physiology app that connects the smartphone with a physiology wristband (BEURER AS 97). This app sends markers (e.g., beginning and end of exposures, beginning and end of bed time) and transfers the physiological data to save local storages at the university hospital. Data quality and entrance of the data into the hospital’s safe databases is continuously supervised and patients are reminded to complete the questions if they forgot it.

### Psychophysiology

To ensure objectivity of data while using a BEURER AS 97 wristband, we will assess heart rate and activity (body movements) to indicate distress levels in the children during one afternoon until the morning for 1 day during the week and 1 day during the weekend [62]. Additionally, we will assess baseline physiology before each therapy session to register distress levels. Activity levels during sleep will estimate sleep quality.

### Manual/therapy sessions

The psychotherapy sessions will be conducted by experienced psychological psychotherapists and they resemble classical in-office psychotherapy with the exception of the usage of teleconferences with the software Vidyo and the additional availability of data for the therapeutic procedure assessed by the app. For the teleconferences, patients will use a tablet and home WIFI and for the app a Samsung Galaxy A5/A50 smartphone with mobile internet provided by the study team. In addition, children will wear a Beurer active AS 97 wristband to measure physiological data. The therapy consists of 14 weekly sessions which each last around 90 min, completed within 16 weeks. The procedure and content of the therapy including material are based on the behavioral therapeutic manual of Wewetzer and Wewetzer [63]. Work sheets will be provided through the cloud BW sync&share; however, this service will soon end for our department. Therefore, a cloud system from our clinic will also be provided whereby each patient and family have their own folder, where they can upload completed material. The treatment will start with psychoeducation, followed up by cognitive interventions and exposure therapy starting in session 4 in combination with response prevention and cognitive therapy. At the end of treatment, there will be two sessions with additional elements for relapse prevention. Each session starts with questions to homework and technical problems. Before each session, the therapist will evaluate the app data from during the week and discuss relevant points with the children and later also the parents. During the session with the child, patient and therapist will work on exposures and cognitive elements. The session will include elements where the children are alone and where the parents are alone and all together depending on children’s age. At the end, there is also some contact with the parents. After every session, patients, parents, and therapists will answer the questionnaires of the session online. More information about the manual can be obtained from the authors. If there are any events that lead to the conclusion that our patients should need another form of treatment, we will lead them to a more suitable treatment option. Any adverse events, protocol injuries, or unexpected events will be noted, followed up by an internal consultation and decision about the subsequent procedure with all members of our team.

### Technical equipment

We will use the home WiFi access for the teleconferences which are provided via the software Vidyo on tablet, display 10.1 inch 1920 × 1200 IPS, CPU: Qualcomm Snapdragon 625, Memory 4 GB, Storage 64 GB, OS: Android 7.1.1 offered and configured by our department. Therapists will use computers with webcam in their offices at the department. Children and parents will also receive configured smartphones with the questionnaires and the physiology app and mobile internet data packages. Therapy materials are provided via the cloud bwSync&share/Clinic cloud. Physiological data is assessed with Beurer AS97 wristbands.

### Data security concept

We will conduct the study in collaboration with the IT department and data security specialists of our university. The data security concept is available from the authors upon request. The smartphones and tablets are secured with passwords and a software to block undesired internet platforms. The password is only exchanged with the families when required because of technical problems. Families have to sign that they ensure that they use the hardware safely only for study purposes and taking care to protect their data. Data of the app will be safely transferred to intermediate servers and from there to servers in the hospital, all encrypted and secured with firewalls. There is no long-term storage of data on the smartphone. After study leave, all devices will be newly configured. An overview over the used Integrated Mobile Health Research Platform (IMeRa) can be found on the following website: http://www.medizin.uni-tuebingen.de/nfmi/imera/imera_start.html. Worksheets and other therapy material will be exchanged between the therapist and the families via the safe clouds bwSync&Share/clinic cloud. Individuals get password-protected folders. All data will be stored using pseudonyms. Apps and cloud folders will be protected from people who are not part of the study team accessing the data. Regular data entry into the databases are continuously checked by our team members. Both apps were developed by the IT department. The reasons for this were data protection and the avoidance of unwanted data manipulation (e.g., extrapolation or smoothing of measured values).

### Statistical analysis

We will use SPSS for data analysis. For all diagnostic parameters, we will assess treatment efficiency with ANOVAs with group as the between-subject factor (treatment group (experimental group), waiting group (control group)) and time (t0 = diagnostic assessment, t1 = end of treatment treatment group/end of waiting period, t2 follow-up I treatment group/ end of treatment waiting group, t3 follow-up II treatment group/follow-up I waiting group, t4 follow-up II waiting group) as the within-subject factors. The most important outcome analysis will be the comparison in CY-Bocs score between the treatment and waiting group at t1. A comparable repeated measures ANOVA will be conducted for the time between t0 and t1 concerning weekly app questions/aggregated physiological data over the week but the time factor is week 1 to week 14. This is to indicate weekly symptom course and differences to the data assessed during waiting in the waiting group. Deeper analyses will concentrate on symptom course and intra-individual variability for daily app questions. Answers of child, parents, and therapist are correlated to investigate their agreement. Additionally, we will evaluate if avoidance behavior during exposures is related to treatment success within exposures with correlations of severity of avoidance behavior and anxiety decrease during exposures. We will also evaluate the influence of avoidance behavior during exposures and during the day on symptom decrease over the treatment assessed via CYBOCs with correlations. Descriptively, we evaluate the answers of our post-experimental questionnaire to treatment compliance, feasibility, and satisfaction as well as the within session questions. We will also compare the number of dropouts between the experimental group and the waiting group with *t*-tests. Integrity is descriptively evaluated with the session questionnaires provided by the therapist. Data will be analyzed to see if missing values are correlated with any basic effects. Little’s Missing Completely at Random Test (MCAR) will be used to check whether they are randomly distributed. The recommendations of the National Research Council will be followed. If there are missing data, we will report analyses with all data available. There will be enough data for the comparison between t0 and t1. A second set of analyses will also include the follow-up assessments where some missing data might appear. We might additionally report analyses with imputations. Significance will be followed up with Bonferroni-corrected paired contrasts. Alpha level is 5% and all tests will be conducted two-tailed.

## Discussion

With our approach, we aim at developing an internet-based state-of-the art psychotherapy for children and adolescents with OCD conducted by psychotherapist experts ensuring a wide acceptability in patients, therapists, and the health system. Previous internet-based approaches in OCD mainly used self-help elements, partially supported by email contacts of psychotherapists, often no experts and partially with small sample sizes [5, 12, 26, 36, 37]. These approaches have many advantages and were very successful; nevertheless, there is indication that the treatment success may be stronger when the therapist is directly involved as summarized in the introduction of this paper. To be able to address the same therapy effect factors as found in classical face-to-face therapies, we used an approach with all elements of a highly effective cognitive face-to-face behavioral therapy and only replaced contacts in the office by teleconferences and enhanced the therapy by real-time information through an app. We are convinced that such an approach would be more acceptable by health insurance providers and also psychotherapists and patients. Since the outbreak of the corona pandemic, psychotherapists have been increasingly forced to use teleconferences to treat their patients instead of face-to-face sessions. During this period, tele-medical approaches are more than ever a high-priority topic.

So far, our experience with our internet psychotherapy of OCD patients and their families is based on our pilot study of feasibility to develop the treatment procedure and the first treated patients in this study evaluating treatment efficiency. Overall, results are very positive with respect to symptom decline, patient, and therapist satisfaction and compliance and we are convinced about the feasibility of our approach.

Although our experience is very positive, there are several points that must be considered when conducting internet-based psychotherapy in patients. An important issue in such study approaches is surely the implementation and development of the data security concept and data transfer which required meticulous thought and work in the current study. Furthermore, the project was developed within intensive discussions in our international OCD experts work group to be able to deliver psychotherapy with such a high quality. Using technology of course also requires an effort by patients and therapists as well as continuous support with respect to technical problems (e.g., no internet connection of the smartphones, data delivery problems, video sessions cannot be started). It also must be noted that mobile internet in Germany has to be improved. Therefore, our app was developed in such a way that data transfer was minimal. Additionally, families are required to be equipped with WIFI access that is, however, mostly realized. It is also important to consider that webcams sometimes have the problem that the therapist cannot see everything in the room, especially with more family members involved. It is also not always possible to see if the patient focuses on the feared object during exposures. Therefore, it can sometimes be helpful to use 180 or 360° webcams or also eye tracker glasses to follow the gaze of the patients. Of course, we intend with our project to detect and to eliminate technological problems and to perfect the treatment manual which will then be easy to use by psychotherapists, including those with less experience of OCD therapeutic interventions. One further important point to be considered is the occurrence of unforeseen circumstances such as a case of emergency during teleconferences, which are beyond the control of the therapist. Then, it is important to contact the ambulance or police.

Finally, while the use of internet approaches is permitted, it is not yet confirmed whether all health insurance providers will pay for this type of therapy, which will impact the acceptability and distribution of these approaches. The Corona pandemic has paved the way for such contracts to begin to establish, perhaps hybrid approaches with both face-to-face and internet-based therapy elements could be the future.

In further studies in collaboration with our iCBTinOCD group, we are planning to develop a multisite individualized stepped care study to investigate which OCD patients need what extent of psychotherapy and also which kinds of therapy, such as classical in-office psychotherapy, internet-based psychotherapy, telephone therapy, or medication. Additionally, we intend to derive internet-based treatment protocols for other psychiatric or somatic disorders with mental problems. Further research is needed to investigate how much therapist contact is needed for internet-based psychotherapies. It will also be important to investigate internet-based treatment effect factors and also side effects [15]. Predictors of treatment success have to be detected, possibly with the help of machine learning approaches. Furthermore, we need more information about long-term effects of these new approaches which will develop over time.

We conclude that internet-based psychotherapies have—beyond all challenges—a lot of advantages and may reach patients that have no other possibility to get competent help, especially in rural areas. With increasing digitalization, these approaches could become more and more prominent and may be able to link different experts struggling to get together for the health of our patients. Therefore, internet-based approaches may become an important further treatment approach besides classical treatment options.

## Trial status

This is protocol version 1. Participant recruitment began in May 2019, and the project will approximately be completed in February 2022. Our active recruitment including a cost-intensive online campaign ended in December 2020. Afterwards, no further patients were included into the study and we will start with data preprocessing of the available patients in December 2021 as the final sample. An earlier submission to *TRIALS* was not possible as the authors were involved in clinical requirements further complicated by the pandemic and the project timeline had to be extended.

## Data Availability

Material and later data can be obtained by the corresponding authors upon request.

## Abbreviations

iCBT: Internet-based psychotherapy
OCD: Obsessive-compulsive disorder
GBIT: Geschäftsbereich Informationstechnologie
IMeRa: Integrated Mobile Health Research Platform
K-SADS-PL: *The Schedule for Affective Disorders and Schizophrenia for School-Age Children Present and Lifetime Version*
*CY-BOCS*: *The Children’s Yale-Brown Obsessive Compulsive Scale*
CGI-S: *Clinical Global Impressions-Severity*
*CGI-I*: *Clinical Global Impression scale- Improvement*
COIS-RC: *The Child Obsessive-Compulsive Impact Scale*
YSR/11-18R: *The Youth Self Report*
CBCL/16-18R: *Child Behavior Checklist*
SCARED: *Screen for Child Anxiety Related Emotional Disorders*
KINDL/Kiddo KINDL: *Questionnaire for Measuring Health-Related Quality of Life in Children and Adolescents*
ULQIE: *Lebensqualitäts-Inventar für Eltern chronisch kranker Kinder*
CFT-20-R: Culture-free test
DIKJ: *Depressionsinventar für Kinder und Jugendliche*
JTCI/JTCI/7-11: Junior Temperament and Charakter Inventar
*HEXACO*: *Personality Inventory-Revised, Honesty-Humiliyt, Emotionality, eXtraversion, Agreeableness (versus Anger), Conscientiousness, Openness to Experience*
CTQ: *Childhood Trauma Questionnaire*
ETI-KJ: *Essener Trauma Inventar für Kinder und Jugendliche*
THQ: *Trauma History Questionnaire*
